# Cryo-Electron Microscopy Studies of Biomolecular Structure and Dynamics

**DOI:** 10.3390/mi15091092

**Published:** 2024-08-29

**Authors:** Arkadiusz W. Kulczyk

**Affiliations:** 1 Institute for Quantitative Biomedicine, Rutgers University, 174 Frelinghuysen Road, Piscataway, NJ 08854, USA; arek.kulczyk@rutgers.edu; 2 Department of Biochemistry and Microbiology, Rutgers University, 75 Lipman Drive, New Brunswick, NJ 08901, USA; 3 CryoEMcorp, Bridgewater, NJ 08807, USA

The technical innovation of the last decade has provided novel tools that are now transforming the field of biophysics by bringing remarkable atomic level insights into the mechanisms employed by bio-micromachines to sustain life. Several novel techniques have been developed that allow for studies of biomolecular structure and dynamics at unprecedented spatial and temporal resolution. One of such techniques, cryo-electron microscopy (cryo-EM), has truly revolutionized structural biology. With recent advances in direct electron detectors and improvements of microscope optics, it is now possible to obtain the so-far unattainable atomic structures of biomolecules that are of paramount importance for basic science and pharmaceutical research. Furthermore, the continuing developments of computational algorithms for image analysis, some of which employ artificial intelligence (AI), and the fabrication of novel instruments for rapid specimen vitrification allow for the detailed structural study of biomolecular populations representing transient intermediate states and molecular fluctuations. The goal of this editorial is to provide a teaser of the growing innovation and complexity in the rapidly expanding field of cryo-EM by highlighting some of the recent research and review articles.

The introduction of direct electron detectors to cryo-EM in the first decade of this century has triggered the snowball effect that led to the determination of thousands of new biomolecular structures. For instance, approximately 99% of membrane protein structures constituting nearly 60% of all currently validated drug targets have been solved within the last ten or so years [1]. To compensate for electron beam-induced movement, modern microscopes equipped with direct electron detectors acquire a series of movies. The first step in movie processing that involves the alignment of individual frames is often conducted concurrently with ongoing cryo-EM data acquisition in real time. Such alignment is detrimental for preserving high-resolution information content for downstream processing. In their research article, Strelák et al. [2] present a comparative analysis of several novel software packages dedicated to movie alignment. The authors examine the quality and precision of alignments performed by each program and evaluate the associated scaling performance. The study provides a valuable practical insight into the advantages and disadvantages of using particular software platforms under varying experimental conditions and computational resources.

Recent technological advancements have made small biomolecules (<100 kDa) amenable to cryo-EM structure determination [3,4,5]; however, experiments often yield Coulomb maps at an intermediate resolution (4–6 Å). The research article by Kelly et al. [6] outlines the computational approach combining rigid-body refinement and simulated annealing to delineate structural conformational variability in such a scenario. The authors applied their newly developed protocol to analyze different conformational states of the nucleocapsid (N) protein monomers and dimers from SARS CoV-2, the virus that caused the COVID-19 pandemic. Although it is often difficult to differentiate between intrinsic biomolecular dynamics and the experimental effects, both of which can in principle deteriorate resolution, the approach introduced by the authors represents an important step toward the development of automated computational methods for deciphering conformational variability of small biomolecules. Kelly et al. conclude that simulated annealing incorporated into real-space refinements holds the promise of elevating model interpretability. In a similar context, DiIorio et al. [7] present a comprehensive review detailing current state-of-the-art methods for cryo-EM data analysis of intrinsically dynamic and heterogeneous samples. The review presents a roadmap for successful high-resolution structure determination of multiple conformers that may be present within the same dataset. The authors start by describing a typical cryo-EM workflow, the process of 3D reconstruction, and an overview of the origins of sample heterogeneity. Next, they review applications of time-resolved cryo-EM (trEM). Of note, recent developments of the custom-built trEM devices designed for rapid cryo-plunging allow for the structural probing of the transient conformational states of biomolecules on the timescale of tens to hundreds of milliseconds [8]. The authors outline various algorithms that aim to resolve discrete sample heterogeneity through 3D classification and refinements, such as, for example, unsupervised maximum likelihood and stochastic gradient descent. In addition, focused and masked classification and multi-body refinements are described. Lastly, the authors review methods developed to resolve continuous conformational changes in flexible biomolecules, for instance, via principal component analysis-based 3D variability, and they introduce novel deep learning approaches. The strength of this review article lies in the practical insight provided by the authors, including an in-depth discussion of multiple test cases and specific recommendations offered for applying each method.

Another review article by DiIorio et al. [9] describes the current state-of-the-art AI-based methods for ab initio structure determination, heterogeneous 3D reconstruction, and atomic model building. The abovementioned steps remain major bottlenecks in a conventional cryo-EM processing pipeline because they demand substantial computational resources and manual intervention. Recently introduced deep learning approaches have the potential to overcome current limitations. The authors summarize the new methods and compare them to conventional techniques while discussing the advantages and disadvantages of both approaches. Different types of neural network architectures are presented, such as, for example, a convolutional neural network, a generative adversarial network, a variational auto-encoder, and other methods utilizing manifold embedding. Specific software packages utilizing the AI methods are presented when appropriate, along with examples illustrating their applications. The review article provides a valuable insight, notably focusing on challenging samples displaying both compositional and conformational heterogeneity. The program AlphaFold2 [10], employing the transformer neural network to process information extracted from multiple sequence alignments, is also described. AlphaFold2, along with the recently introduced AlphaFold3, have already demonstrated an unprecedented level of accuracy in modeling and predicting protein structures with sub-angstrom precision. They have become widely used tools in multiple fields. Importantly, the review article points to the areas where future developments are needed, for instance, by highlighting the lack of a physically interpretable metric that would explicate molecular trajectories linking different conformational states of biomolecules computed using the manifold techniques.

Breakthroughs in the field of cryo-EM spearheaded developments of several related technologies. Noteworthily, cryo-electron tomography (cryo-ET) and subtomogram averaging have recently emerged as powerful imaging techniques that enable direct 3D visualization of intracellular structures in their native environment at the nanometer or higher resolution [11]. The limited penetration depth of biological samples by electrons and image blurring due to inelastic scattering necessitate relatively thin samples of approximately 1 µm in thickness. Recently introduced focused ion beam-scanning electron microscopy (FIB-SEM) overcomes this bottleneck, as it allows to reduce the thickness of the vitrified samples to tens of nanometers while preserving the intracellular structures of interest and making them suitable for the subsequent cryo-ET analysis. Another important application of cryo-ET is its integration into a correlative light and electron microscopy (CLEM) workflow, in which fluorescently labeled structures of interest are investigated using light and electron microcopy while providing insights into both biomolecular structure and dynamics [12]. Given the unpresented expansion of cryo-EM and its derivative methods, many of which could not be presented in this editorial due to space constraints, it is safe to predict that the field will undoubtedly continue to flourish while unveiling intricate modi operandi of biological micromachines in the years to come.

Last but not least, as the Guest Editor of the recently published Special Issue of Micromachines on Electron Microscopy and Single Molecule Studies of Biomolecular Structure and Dynamics, I would like to take this opportunity to thank the authors for contributing their articles and the reviewers for taking the time to improve the quality of submitted manuscripts. My special thanks to the members of the Micromachines Editorial Office and particularly to the Assistant Editor, Dr. Nasuha Binte Rohaizad, and the Section Managing Editor, Mr. Xin Yuan Thow, for their support in the management and promotion of the Special Issue.
